# Defining the Characteristics of an e-Health Tool for Suicide Primary Prevention in the General Population: The StopBlues Case in France

**DOI:** 10.3390/ijerph20126096

**Published:** 2023-06-09

**Authors:** Anaïs Le Jeannic, Kathleen Turmaine, Coralie Gandré, Marie-Amélie Vinet, Morgane Michel, Karine Chevreul, on behalf of the PRINTEMPS Consortium

**Affiliations:** 1 ECEVE, UMR 1123, Université Paris Cité, Inserm, 75010 Paris, France; 2 Unité de Recherche Clinique en Économie de la Santé (URC Eco), Assistance Publique-Hôpitaux de Paris, 1 Place du Parvis Notre-Dame, 75004 Paris, France; 3 Unité D’épidémiologie Clinique, Assistance Publique-Hôpitaux de Paris, Hôpital Robert Debré, 75019 Paris, France

**Keywords:** e-health, application, mental health, suicide, primary prevention, general population

## Abstract

With over one million deaths per year in the world, suicide is a major public health problem that could be significantly reduced by effective prevention programs. E-health tools are of particular interest for primary prevention as they can address a broad population including people unaware of their own risk and provide information and help without the fear of stigma. Our main objective was to define the overall characteristics of an e-health tool for suicide primary prevention in the French general population by defining the characteristics of the IT features; the content of the information delivered; the best way to structure it; and how it should be relayed and by whom. The research was carried out through a literature review and a co-construction phase with stakeholders. Four types of strategies may guide the construction of e-health tools for suicide primary prevention: education and awareness, (self-)screening, accessing support, and mental health coping. They should be accessible on different devices to reach the most users, and language and content should be adapted to the target population and to the issue being addressed. Finally, the tool should be consistent with ethical and quality best practices. The e-health tool StopBlues was developed following those recommendations.

## 1. Introduction

### 1.1. Background

With almost a million deaths per year, i.e., one death every 40 s, and at least twenty times as many attempted suicides, suicide is a major public health concern [1,2]. This has led many governments to try and address this matter [3,4,5,6], as nearly half of people with suicidal thoughts do not receive any form of care in high-income countries [7]. Two main reasons may explain this at the individual level: individuals at risk of suicide do not recognize their own risk and therefore do not seek care, and even when they are aware of their suffering, they may be reluctant to express their needs, either preferring to handle the problem alone, believing that spontaneous recovery will occur, or thinking that the problem is not severe or that no treatment would be effective [7,8]. People may also be reluctant to seek help because of fear of stigma [9] or even self-stigma, as mental illness is still perceived as being related to violence and abnormality and leading to social rejection in many countries [10,11,12,13,14]. In fact, up to 90% of suicides occur in people suffering from mental disorders, with more than half having a depressive disorder [15]. Other suicides may also have an underlying psychiatric problem [16]. Thus, if not a disease by itself, suicide can be a symptom of many pathologies, and may therefore have been prevented if these individuals had received timely and appropriate care. 

Yet, despite suicide being a cause of avoidable mortality and morbidity that could be significantly reduced by effective prevention programs, few measures have been implemented apart from restriction to lethal means. Additionally, suicide prevention remains insufficient in relation to needs [17,18,19,20] despite showing effectiveness when implemented at a national level [5]. Regarding primary prevention, telephone helplines are currently the only large-scale option available for the general population in many countries including France, but they are not able to answer all calls due to overwhelming demand [21]. Moreover, those types of measures do not address the main challenge in the field of suicide prevention, i.e., the impaired upstream identification and care for people at risk, with the World Health Organization (WHO) singling out early detection and management of mental disorders and suicide ideations as one of the key priorities for efficient suicide prevention [22]. Results from the French National Health Data System (SNDS) between 2013 and 2015 [23] show that people who consult for a mental health condition account for 37% of suicides and that many of them use the healthcare system: two-thirds the month before death, and one-third the week before, with most of them visiting a GP or an emergency department. Primary care then appears to be the right place to implement this upstream detection. In addition, a better recognition of mental health needs would help identification, especially for men, as it raises their use of mental health services to match that of women [24].

In this context, e-health—the use of information and communication technologies (ICT) for health [25]—and its subbranch mobile health (m-health) could be one way to achieve this goal. Indeed, internet programs can target a broad population, including people with psychological distress who are not aware of their own risk of suicide, thereby enabling the development of primary prevention programs. As such, they may help develop self-recognition of suicidal risk and associated mental disorders as well as to improve help-seeking behaviors in the general population. Another advantage of e-health programs available through smartphones and computers is that they can be accessed remotely and at no additional cost to the user [26], including among socially-isolated and vulnerable individuals who are otherwise hard to reach [27,28]. They also provide the opportunity to look up information and help without fearing stigmatization, as those tools can be used anonymously. 

However, it is necessary to improve access to evidence-based information as some of the materials available on the Internet present high levels of inaccuracy [29,30,31]. Over 10,000 mental health- and psychiatry-related smartphone apps are currently available, mainly in English, and that number is still growing, but they are rarely evaluated [32]. In addition, many options are available in terms of IT features, software engineering, type of media, etc., and another challenge is to make the right choice of tool and content to develop an intervention best suited to the diverse needs and situations of people at risk of suicide. Likewise, the way in which the information is relayed (text, videos, pictures…) and by whom (professionals, peers…) can make an important difference in how it is understood and therefore how helpful it will be [33].

Thus, even if e-health tools could be seen as an effective opportunity to inform and detect people in need of mental health care, no guidelines are available on the characteristics that those tools should fulfil to properly respond to the current need. Moreover, it is now well established that co-construction through participative research is important, as it improves the quality of the research itself, but also enhances how its results will be perceived by stakeholders [34].

France is particularly in need of an effective tool for suicide primary prevention as it has one of the highest suicide rates in western Europe (17.9 per 100,000 inhabitants in 2016 after standardizing for age [35]), and each year over 10,000 individuals take their life and around 200,000 attempt suicide [36]. Secondary prevention remains difficult except by educating carers and population to the signs of the suicidal crisis [37], and tertiary prevention is more and more implemented, with brief contact interventions such as the VigilanS program [38]. Suicide primary prevention using e-health tools in France focuses on specific methods to help people with psychological suffering, such as mindfulness (e.g., Petit BamBou) [39], or with specific conditions such as depression (i.e., Happli Day, no longer available) [40], but proposes no comprehensive, evidence-based and evaluated e-health tool targeting people at risk in the general population. In this context, a research project was funded to co-construct and evaluate an e-health tool for suicide primary prevention, StopBlues (SB) (VO2 Group, France).

### 1.2. Objectives

Our main objective was to define guidelines regarding the characteristics of an e-health tool for the primary prevention of suicide in the French general population. Intermediate objectives were to define (1) the characteristics of the IT features (application with or without an associated website, adaptive website, or WebApp); (2) the content of the information that should be delivered, i.e., which main issues should be addressed to help the target population; (3) the best way to structure that information so it is easy to access and attractive for all users; and (4) how the information should be relayed (e.g., using written or audiovisual formats) and by whom.

## 2. Materials and Methods

The research was carried out in two phases between 2014 and 2017: (1) a review of the literature and of experience was conducted to gather knowledge on existing interventions that may already have proven their effectiveness, and on guidelines for suicide prevention programs; and (2) a co-construction phase was carried out through a participative process (focus groups and interviews) with stakeholders, including the target population, to ensure that the needs of individuals would be met.

### 2.1. Review of the Literature and of Experience

The objective of the review was to identify existing suicide prevention interventions, e-health tools in mental health and more specifically in suicide prevention, and guidelines on what is essential for a successful and ethical intervention in mental health.

#### 2.1.1. Literature Review

The literature review included documents published between 1 January 1989 and 31 December 2016 in French and English from three sources. First, a review of the scientific literature was carried out on Embase, enriched with Scopus and Medline, and combined two groups of keywords: a first group linked to the concept of suicide and suicidality (suicidal acts, behaviors and ideas, self-harm) and the second linked to interventions and prevention programs (Appendix A Table A1). In order to narrow the selection of articles on suicide prevention programs based on the use of technological tools (websites and smartphone applications), an additional search focused on two groups of keywords as filters: the first related to the concept of suicide and suicidality (suicide; suicidal acts, behaviors and ideas; self-harm; intoxication; suicide attempt; mental disease; schizophrenia; suicidal behavior; drug intoxication; depression and major depression), and the second related to interventions and prevention programs accessible online on websites or on mobile applications (computer; telephone; mobile phone; computer system; camera; film). The initial selection of potentially relevant publications was made independently by two researchers based on titles and abstracts. In case of disagreement, the researchers exchanged their views until a consensus was reached. Articles for which there was doubt were included for verification in the next step. Researchers then read the full text of the articles selected during this first selection phase. Retained articles were those containing information and recommendations on suicide prevention, mental e-health tool development and content.

#### 2.1.2. Review of Experience

A review of experience of e-health research projects focused on prevention in mental health and suicide was also conducted at a European level on Google Scholar to look at how European countries had approached the issue with their own prevention projects and what recommendations had resulted from those projects. In fact, the definition of mental health has changed over the years; with depression and suicide now being globally recognized problems, and with more and more countries working on their prevention, many projects on mental health prevention have been financed by European funds through the Seventh Framework Program (FP7) [41].

Websites, applications, and news articles related to e-health, notably mental e-health, and suicide prevention were also investigated in an international review of experience from French- and English-speaking countries between January 1989 and October 2016 by implementing alerts (on Google and Google scholar) with the same keywords as above. The investigation was conducted throughout the co-construction phase (until spring 2018) so as to stay up-to-date with the fast-growing market.

### 2.2. Focus Group

#### 2.2.1. Rationale

Among participative research methods, focus groups (FG) are a particularly useful tool, allowing participants to exchange opinions and experiences. This creates a group dynamic that can lead the research in new directions [42,43,44]. Overall, FG has three main advantages. First, it provides an opportunity to obtain the opinions of potential e-health suicide prevention tool users and mental health practitioners, and to encourage critical discussion. Second, it encourages the involvement of stakeholders by providing them a voice and taking their experience into consideration. Finally, it provides researchers the opportunity to develop projects that meet the expectations expressed by the people concerned [34,42,43].

#### 2.2.2. Participants

Our FG participants were selected using purposive sampling rather than random sampling as the latter would have been unlikely to represent the whole population of users and our goal was to target the general population [45], and because participants needed to have specific knowledge and experience about suicide prevention, psychological suffering, electronic devices, and research interventions to ensure the richness of the exchanges [46]. A variety of stakeholders, namely, representatives of potential users, as they may have more insight and experience than random users; health professionals, to have the point of view of caregivers used to dealing with people with psychological distress; and IT specialists, to help us stay realistic regarding the tool itself and propose options not considered due to unfamiliarity of those not in the technical field, were all included in the same sample. Indeed, there was no reason to separate the different stakeholders as they were expected to share and discuss their opinions and ideas [45]. 

Regarding the number of participants, each category had to be represented and the final number of participants could not be too high to allow for harmonious dialogue; therefore, a sample size between six and ten participants was chosen. Participants were contacted through the network of the research team. Public health researchers were present during the FG as mediators and note-takers [47].

#### 2.2.3. Procedure

A structured interview guide based on the literature review and structured along each intermediate objective was used. The first session began with members of the research team presenting the main results of the literature review and the purpose of the FG. Each following session began with a reminder of the main discussions and propositions of the previous one to ensure that everyone agreed. Remaining issues were then discussed one after the other between the participants, with guidance from the moderators to stay focused on the subject and afford everyone the opportunity to speak [44]. 

Iterative sessions took place until a consensus was reached on a clear and structured base for the e-health tool. The discussions were audio recorded (with the agreement of the participants) and transcribed synthetically. 

#### 2.2.4. Analysis

After each session, a thematic analysis was carried out manually by two independent researchers, focusing on the ideas that were raised several times by the participants to identify repeated patterns of meaning, with categories and concepts identified and organized around main themes [48]. After confrontation of the findings from both researchers and synthesis of results, a team meeting was held to discuss the results and assess the need for additional sessions. Finally, a global analysis of all sessions was carried out to synthesize the main recommendations.

### 2.3. Experts’ Interview

Clinical and technical issues arose during the FG that could not be resolved immediately, such as final choice of questionnaires. Interviews with psychiatrists and IT specialists were organized by mail and/or phone to get additional information and guidance [49,50,51]. Those interviews were completed using a supplementary literature review regarding the corresponding issues to complete the information provided by experts. 

## 3. Results

**Figure A1:**
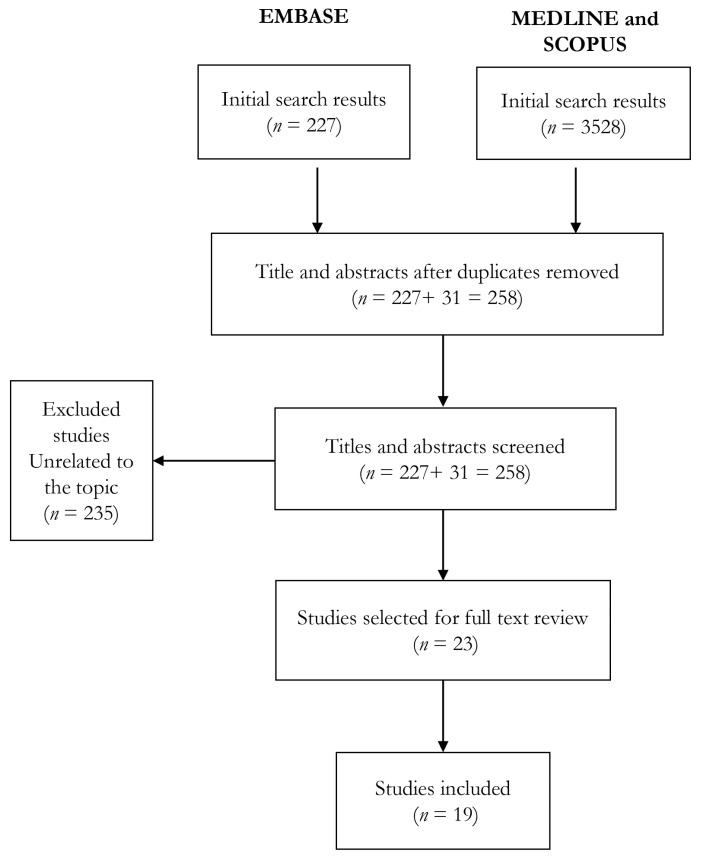
Flowchart of literature review.

A total of 258 scientific articles were selected based on title and abstract, and 19 of them retained after full reading by two researchers (Appendix A Figure A1). Four programs were identified for their recommendations on how to establish an evidence-based program in mental health prevention, with three European programs regarding e-health prevention programs [52,53,54,55,56,57], and an Australian program targeting global suicide prevention [58,59] (Table 1). Three applications for suicide prevention were selected for detailed analysis because they were evidence-based, showed some efficacy on suicidal ideation [60], had positive feedback from users at risk [61] or were award-winning [62], and gathered the main functionalities developed by the other e-health programs reviewed. Detailed characteristics and advantages of the applications are presented in Table 2. 

Three FG sessions were held until a consensus was reached on the IT features of the tool and its content. The mean number of participants was eight, out of a panel of 11 stakeholders, depending on their availability. Each session was composed of at least two psychiatrists working in the field of suicide, a psychologist with experience developing e-health tools, two IT specialists, and a patient association’s representative. In addition, three of the participants were contacted individually as part of experts’ interviews (see Section 2.3). 

Combining the results from the literature review, the FG, and interviews, the overall characteristics of an e-health tool for suicide primary prevention were defined as follows, and were then applied to develop the tool called StopBlues (SB).

### 3.1. Characteristics of IT Features

IT features correspond to the digital characteristics of the e-tool and include the type of device it is intended for (smartphone, tablet, or personal computer), the type of application (native app, WebApp, website) and its access conditions, and the operating system (OS), as well as security and storage management regarding users’ data and ethical dispositions to ensure quality content for users.

Regarding the digital characteristics, the FG concluded that an e-health tool for primary prevention should have high population exposure and that users should have access to all functionality even when offline, as a moment of anxiety or unwellness may occur anytime, anywhere. Consequently, a native application was preferred to a WebApp, as it is not dependent on an internet connection. Thus, poorly connected populations are not excluded, and being in a place without an internet connection is not a barrier when in crisis. Because access to the internet varies across social classes and ages [67], the tool should be accessible on both mobile and computer devices, to reach all types of users with a continuity of service. A sister website should therefore also be developed for those connecting on computers only. In addition, the tool should be developed for both OS Android and iOS as only targeting the former would exclude around 23% of users [68]. Finally, there was also a consensus among the FG participants that any prevention tool created with public research funds should be accessible free of charge to all, so as to not exclude vulnerable or deprived individuals. 

In accordance with those findings, SB is thus composed of a native application and its sister website that contains the exact same content. It is provided for free on Android and iOS systems.

Data security being a priority, as stated in the Euregenas guidelines [54], and because the collected data may be sensitive individual data, all collected data should be stored in a certified health data hosting service [69], as is the case for SB users’ data.

From an ethical standpoint, it is the duty of public institutions to propose evidence-based tools that do not endanger users. It is then essential to pay particular attention to the content offered to users who are in psychological distress and may be fragile and trusting. App quality indicators [30] can help the tool reach a certain standard consistent with best practices, which is a pledge of quality for users: stating by whom it was developed, how to contact the creators if needed, what the references for the source material are, having users read and sign a privacy policy to protect them and developers, and assuring users with a final layer of protection by way of personal identification. 

**Figure A2:**
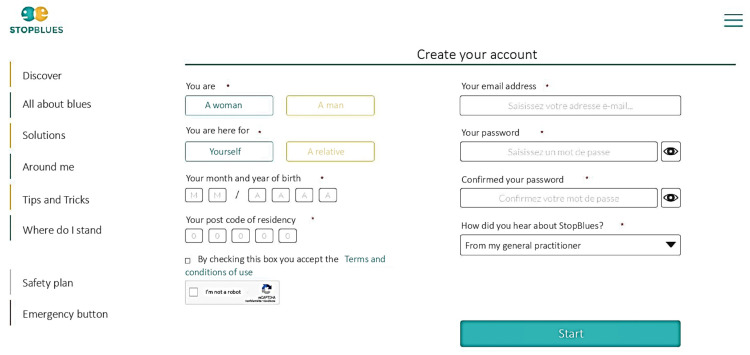
Screenshot from the website: collection of personal data during subscription.

In SB, the status and name of the creators are explicitly shown, a privacy policy is included, and users’ accounts are protected by a username and password. Moreover, users have to read an information note and provide consent before subscribing to SB (Appendix A Figure A2). While there are no references provided, it is clearly stated that the entire content is issued from a research process and is evidence-based, and all contributors are identified by name and profession. 

### 3.2. Content and Structure of the Information

Several suicide prevention strategies have been described in the literature [17,30,70] but not all have been evaluated with high levels of evidence. Four of them were retained by the FG to be included in the tool. 

#### 3.2.1. Awareness Strategies to Improve Knowledge in the Target Population

Both the literature and the FG pointed out that many false assumptions such as “depression is a weakness” and stigma around mental health lead to people being in denial or persuaded that they can manage it on their own [7,8]. It has also been shown that increasing knowledge on mental health issues reduces self-stigma and stigma, and therefore leads to an easier process to self-recognition and help-seeking [9,71]. In fact, the earlier the disorder is treated, the easier it is to manage and even recover from [72]. The same applies to suicide and clinical situations that can lead to the latter, among which are depression, anxiety, and substance abuse [73,74].

That is why the information delivered to users of a suicide primary prevention tool must help them overcome denial of their mental health impairment, acknowledge their situation, and, if needed, seek help from the appropriate people. In particular, information should be provided to help people better understand what can lead to the suicidal act and how to stop the process. 

Different themes should be proposed to users. First, what the causes and risk factors for poor mental health are, as well as the signs that can help identify it in oneself or in others. Preconceived notions about mental illness and suicide have to be deconstructed to inform people that mental illness is not a weakness and can affect everyone. The main treatment options making consensus should also be presented, depending on the clinical situation (emergency or not), what they can provide, and the user’s level of acknowledgment of his or her situation. This should include persons to whom users can talk (relatives, therapists, and doctors), the structures they can go to for care (emergency departments, psychiatric ambulatory care centers, etc.), and the different therapies and medications (psychotherapies, positive psychology, antidepressants, etc.) and how they can help them. 

All the points raised above should be addressed for the tool to be complete and effective. In order to do so, the information in SB is structured around two axes. The first one, called “All about blues”, describes mental health issues in four subsections: (1) a description of the signs of mental health disorders; (2) their possible causes and risk factors; (3) details on the fact that poor mental health is not a weakness and can affect anyone; and (4) a focus on suicidal thoughts, suicide attempts, and suicide. The second axis provides information on “Solutions” to these issues in three subsections: (1) encouraging people to talk about their psychological distress and seek help; (2) an explanation of the different forms of treatment available by mentioning recognized therapies and medications; and (3) a presentation of different ways to self-maintain, restore or improve mental health, such as practicing physical activities, positive psychology exercises (yoga, meditation, etc.) [75,76], and other coping tools to choose from depending on one’s own preferences. 

Moreover, operating within the field of suicide primary prevention implies helping users from the early stages of their questioning about not feeling well to accompanying them while in crisis. That is why the concept of suicide should be treated carefully and separately, as it is a sensitive subject and not all people in psychological distress are at risk or even realize it could be a risk. Mentioning it too often or mixed up with other concepts could repel some users and dissuade them from continuing to use the tool, by frightening them or leading to think it is not appropriate for them.

#### 3.2.2. Self-Screening Strategies to Improve Recognition of Suicide Risk and Self-Awareness among the Target Population

As mentioned above, people with mental health issues may be in denial or think that they can manage without help [7,8]. Self-screening can make them aware that there is a problem and help them overcome denial by testing themselves and having a more objective view of where they stand. This process is not a consultation or formulation of a diagnosis but can help people realize they are at risk of worsening mental health or even suicide and raise motivation to seek formal or informal help [71,77].

Self-screening is thus an essential functionality in a primary prevention tool. Self-screening questionnaires must be evidence-based, few and short so as not to overburden users. They should assign a final score along with providing feedback and recommendations, and be suitable for self-administration. Questionnaires should also be available to people seeking information for a friend or relative, to help the user screen the mood and behavior of someone else, in order to be either reassured or guided on how to express their worry. Finally, a suicide primary prevention tool asking about suicidal ideation first would potentially frighten users, in particular if they are at the beginning of their inner process or far from having suicidal thoughts [78]. It is thus important to separate suicide questionnaires from others or activate them when results from the other questionnaires have raised concern.

**Figure A3:**
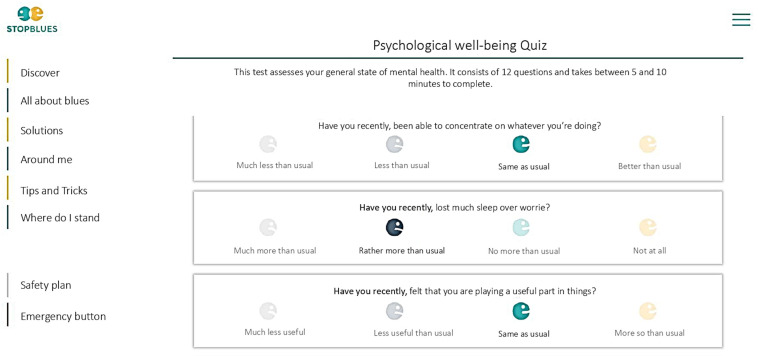
Screenshot from the website: the “Psychological well-being Quiz” from “Where do I stand”.

The self-screening section of SB, “Where do I stand?”, proposes four validated questionnaires: the GHQ-12 to assess global mental health [79,80], the PHQ-9 [81,82,83], and the GAD-7 [84,85] to evaluate depression and anxiety, respectively, and the suicidal risk section of the MINI [86,87] (Table 3). The latter becomes available only if the scores at the other questionnaires reveal a nonoptimal state (GHQ-12 ≥ 2, or PHQ-9 > 4, or GAD-7 > 4). A single questionnaire (MADRS) [88] screening for depression was selected for those subscribing for someone else and who had specified as such upon subscribing. To increase acceptance and make reduce the feeling of filling in a medicalized questionnaire, all questionnaires were entitled “Quiz” and their names were changed to Quiz for psychological wellbeing (GHQ-12) (Appendix A Figure A3), Quiz for depression (PHQ-9), and Quiz for anxiety (GAD-7).

They are presented to users with a short explanation on the mental state they are screening for, the number of questions, and an estimate of the time required to fill it out. A final score and its interpretation appear after filling out each questionnaire. Once all questionnaires are answered, global feedback is provided to users, depending on their scores and evolution over time. If needed, they are redirected to the appropriate feature of the tool or advised to consult. A neutral tone is used to avoid a condescending or paternalistic user experience.

Daily self-monitoring and mood tracking can also help raise self-awareness and have an effect on regulation of emotions with an impact on depression and anxiety [78,79]. It is an easy and playful way to follow daily mood and have a global view of its evolution. A prevention tool can thus benefit from this feature, which can be used to monitor users’ status on multiple aspects: level of anxiety, energy, suicidal ideation, etc. Those differences make them complementary to questionnaires. 

**Figure A4:**
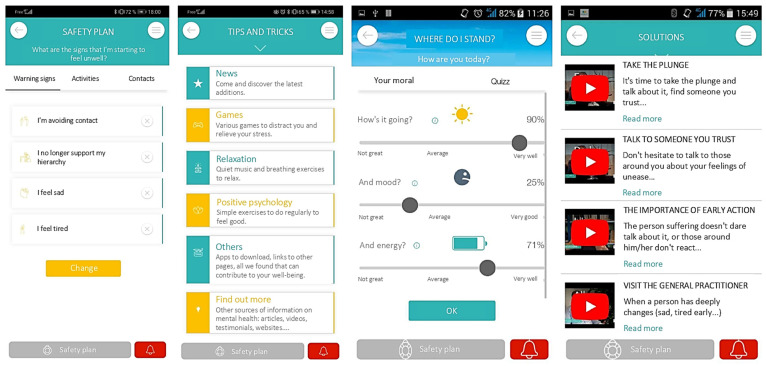
Screenshots from the app: “Safety Plan”, “Tips and tricks”, mood tracker scales from “Where do I stand” and “Solutions’.

It was decided in the FG that visual analogue scales (VAS), with the help of icons, was an optimal way to present them to users. In SB, users can take note of their mood status daily using a mood-tracking system made up of four VAS ranging from 0% (worst possible) to 100% (best possible), rating the user’s global spirit, mood, level of energy, and suicidal ideation (from none to a lot) (see Appendix A Figure A4). Here too, the suicidal ideation scale is proposed only if two other scales drop under 20% or if questionnaires’ scores reveal a nonoptimal state (GHQ-12 ≥ 2, or PHQ-9 > 4, or GAD-7 > 4, or suicidal risk evaluation from the MINI). After validation, a chart appears with the level of each scale in a different color and its trend over time, accompanied by feedback redirecting users to other functionalities of the tool or to help resources if needed.

#### 3.2.3. Accessing Support Strategies to Improve Access to Help and Treatment for Those Who Need It

People experiencing psychological distress may not always be able to handle it by themselves. It is therefore important to provide information on who to contact in that case. The information must be clear, reliable, and all in one place so that people who are not able to look for it because they are psychologically exhausted may find it easily [78,89]. Indeed, information is one of the pillars used in e-health to empower users [90].

Resources information in a suicide prevention tool should help users figure out who can help them, depending on their localization, level of psychological distress, and whom they feel comfortable opening up to. That help can be informal (relatives, friends, clerics, etc.) as well as formal (GPs, psychologists, psychiatrists, etc.). As the former is people-dependent and cannot be controlled, the tool should provide information on both but focus on the latter to inform users who are willing to seek professional help.

There was a consensus in the FG that the best option was to provide users with an interactive map of available help resources. It should be geolocated to show the nearest help resources around them, with filters for each kind of resource (ED, GPs, psychiatrists, etc.), so that users may choose, depending on the level of urgency, what is available nearby and whom they feel ready to talk to [7]. The filters should be determined by the organization of mental health care in the country where the tool is being developed.

The access support section “Around me” is one of the key features of SB and was built following the above recommendations. It is a geolocated map displaying local resources, which are proposed in three main subsections: general health professionals (GPs and hospitals), health professionals providing psychological support (psychiatrists, psychologists, psychiatric emergency departments, and psychiatric ambulatory care centers (centres medico-psychologiques—CMP), and local organizations. To identify the latter, local authorities that were part of the research project and willing to do so provided information on organizations located within their territory. These organizations were mainly associations, either offering mental health support as a main goal or only as a backing for other kinds of help, and other non-associative local structures that could also bring psychological support to someone in need. 

#### 3.2.4. Mental Health Strategies to Improve Prevention of Psychological Distress and Suicide

Cognitive impairment in depression or anxiety may limit people’s ability to act in everyday life [91]. An option identified in the literature to overcome this is to provide the patient with a “toolbox” [78,92,93] with different functionalities and coping techniques at hand to help them cope with their feelings without requiring too much thought or energy, which they may not have the capacity for [93]. The e-health tool should therefore provide people with different evidence-based tools to empower them when experiencing difficulties.

In a suicide prevention e-health tool, users must have an emergency button that allows them to call the appropriate emergency number with just one click to deal with a suicidal crisis (emergency services or a national crisis support center). This button must be accessible at all times, as highly recommended by the ethical guidelines for technology-based suicide prevention programs [54], which means being on every page and in a bright color. 

Another important functionality reported by the literature is the safety plan (SP) [94,95], to be used in case of suicidal crisis. Objectives of an SP are to avail to users all the information they need to handle a suicidal crisis through six sections: (a) warning signs of a suicidal crisis to come (e.g., feeling hopeless, drinking more), to help users realize a crisis is approaching and needs to be addressed with the other sections of the SP; (b) internal coping skills, i.e., strategies to manage a crisis on their own in order to feel less vulnerable (e.g., listening to music, gardening); (c) social contacts and places for distraction, i.e., activities involving other people (e.g., bowling with friends, shopping); (d) nonprofessional support contacts who could help them cope with a crisis (e.g., parents, siblings, friends); (e) professional care contacts (e.g., GP, psychologist); and (f) help to reduce access to lethal means of suicide (e.g., asking for help to store a firearm, locking up medications they would consider using during a suicidal crisis). The SP is filled by the user outside of a crisis, if possible, with the help of a therapist, and is personalized to take the user’s experience, preferences, and contacts into account.

Finally, a “Hope box” with different coping and distracting resources would allow users to find the most appropriate way to deal with a difficult moment through distraction, relaxation, and coping propositions, and is complementary to other functionalities. It allows an adaptation of the tool to different levels of suffering and sensitivities, with the use of different media (e.g., audios, videos, games, pictures, quotes) [92].

In SB, the “Emergency button” is accessible to users on every page in bright red and the national emergency number (112) by default calls. When the user is logged in, it can be personalized with other phone numbers chosen amongst family, friends and care providers who can help handle a crisis situation. On the mobile application, the button allows a direct call to the chosen number by just clicking on it.

A safety plan is proposed in the section “Support plan”, which is available when the user is logged in. It was decided to reorganize the six sections into three to simplify its use: (1) “Warning signs” of a crisis upcoming (see Appendix A Figure A4), (2) “Activities” with solutions (internal skills and distractions), and (3) “Contacts”, i.e., who to contact during a crisis. Moreover, as an SP is usually generated in collaboration with a physician, the tool offers many suggestions regarding signs and coping strategies to help users should they fill it out alone [30,96].

A Hope box, called “Tips and tricks”, proposes everyday coping strategies, such as relaxation, yoga, positive psychology, and mindfulness exercises, to help users manage distressing times on their own (Appendix A Figure A4), as these have been shown to be effective on depression, sleep, wellbeing, and other disorders related to psychological distress [75,97,98]. 

### 3.3. Structure

An e-health tool can be structured with up to three parts: a passive one proposing information generally accessible to all, an active one requiring an active involvement from users (e.g., answering questionnaires), and an interactive one allowing contact with peers or professionals (e.g., using a forum) [30]. The passive part can also be a gateway to the rest of the tool and a showcase to motivate users to go further and log in if needed. The active part may be more stimulating for users as it requires input but can also be seen as repellent to them if it requires creating an account. However, an account should be seen as a beneficial tool as it allows storage of users’ data so that they may have access to the evolution of their answers and SP over time [78]. The account creation step is also a way to have the user choose between different experiences: men or women, youth or adult, patients or physicians. Finally, the interactive part can be the first step to talking to someone [30], in complete anonymity. However, it requires a professional presence for moderation, advice, or consultation [30].

It was decided in the FG that the SB tool would have a passive component with information and an active component that would require users to create an account and log in to access other functionalities. This allows users to choose between two experiences, for themselves or for a relative, and to personalize their profile. 

The addition of a forum or a chat to SB was discussed but not retained, as it would have required professional moderation 24/7, the cost of which was too high regarding the scope of the project. 

### 3.4. Delivery of Information

Constraints exist regarding how the information should be delivered by the e-health tool. Targeting the general population implies adapting messages to all genders, ages, social statuses, and literacy levels [99], and therefore is required to remain neutral and reach a certain balance in messages, neither too alarmist nor too reassuring, while providing enough information that it appeals to most people. 

Moreover, as the target population could be exhausted, depressed, anxious, or affected by cognitive impairment and their mind flooded by their own issues [91], it is important to be careful not to overwhelm users with numerous information and functionalities they cannot handle. Finding the right vectors of information can therefore be challenging. FG participants agreed with the literature on the fact that short videos are an ideal vector of information for two reasons: it makes information more accessible to people with low literacy levels [100] and to people with psychological impairment who are less able to concentrate for long periods of time. Nevertheless, it can also be supplemented with written information, short and to the point.

Two strategies were retained to address stigma in the e-health environment: education (on preconceived ideas) and contact (with a person with mental illness). Regarding contact, emphasis is to be placed on testimonies, as self-identification is a good way to fight stigma [33]. Testimonies should be from different genders, ages, ethnicities, and experiences to maximize the possibility of identification. Testimonial videos should alternate with the educational side, which can be provided by expert interviews bringing a scientific guarantee and clear explanations regarding themes such as depression, treatments, sleeping disorders, etc., and sometimes experiences with some of their own patients [33].

Communication in SB follows those recommendations, with information in short videos of testimonies and experts, a short abstract of their content, and neutral feedback messages after each questionnaire. It was also considered that the suicide theme should be mainly restricted to a single section, and the word used very rarely, contrary to mental wellbeing or unhappiness. 

## 4. Discussion

E-health tools for suicide primary prevention in the general population can be constructed around four types of strategies: education and awareness, (self-)screening, accessing support, and mental health coping. Tools should be accessible on mobile devices as well as computers to reach the most users, and language and questionnaires should be adapted to the target population and to the issue being addressed. In particular, the notion of suicide must be handled carefully. Finally, tools should be consistent with ethical and quality best practices, and data stored in or by a certified health data hosting service.

The structure of the tool should allow users to find the resources they need easily, with evidence-based information being at the forefront to increase users’ knowledge about mental health, explain distress signs as well as solutions for the users themselves or for a relative. Users may then engage in self-recognition and evaluation with the help of validated questionnaires and follow their evolution over time with playful VAS. If needed, users may consult available help resources around them on an interactive map with filters. Finally, at any stage of their evolution, they should have access to a “toolbox” of coping strategies adapted to their levels of suffering and sensitivities, from the everyday “Hope box” to the safety plan to manage a crisis and including the essential emergency button. 

Following these guidelines, SB was developed as a comprehensive e-health tool suitable to a large part of the adult population affected by mild-to-severe psychological distress. It is evidence-based and targets some of the key challenges in the field of psychological distress and suicide prevention such as stigma, self-awareness, and help-seeking behaviors through a large panel of functionalities.

By updating the literature and experiences on the subject throughout the co-construction phase, we made sure to build a relevant tool. However, this review is now five years old and newer e-tools were not included. As this is an expanding field, new mental health applications are now available in French [101]. However, to the best of our knowledge, there are still no existing guidelines regarding development of e-health tools for suicide primary prevention.

Due to financial or time constraints, it is not always feasible to include all functionalities in one tool, and only the most essential are presented here. For example, an interactive part can be an interesting function to include. Indeed, exchanging with peers is very important to overcome isolation and denial [102], for young people in particular [103]. However, a subject as sensitive as suicide would require constant moderation of the chat or forum by trained professionals, which is costly. Many additional ideas emerged from the literature review and FG and were not presented here but could change users’ experience for the better, such as adding subtitles on videos for hearing-impaired users, having a printable safety plan for people only using the SB website, a real personalization of the interface (background, colors, etc.), or even adding a non-player character (NPC) that could be a real plus when accompanying users to help them stick with the application. A complexification of the tool’s algorithm could also enhance user experience, e.g., by presenting users information according to their level of risk assessed using questionnaires. While the objectives of the tool may make those supplementary functions important to include, it is essential not to underestimate associated development time and costs.

Participative research is one of the strengths of this project and has allowed the development of a practical tool as close to what potential users would expect and need as possible by providing them with a voice [47]. Different stakeholders were involved: user representatives, psychiatrists and psychologists, but also IT specialists, creating a group dynamic with complementary and argumentative interactions [104].

It is nevertheless important to note that if the initial goal of the FG was to provoke a positive emulation from the different perspectives [42], the research team was confronted with a situation where some participants were more “dominant” than others, e.g., psychiatrists were more likely to impose their views [44]. This would suggest that a separate FG with only user representatives could have been more appropriate to allow them to express their own point of view more easily [34]. In addition, involving users themselves rather than representatives from patients’ associations would have been interesting but challenging as well, as this implies working with fragile people with a history of mental disorders. This could prove difficult to handle for a team not trained for it and could lead to hurt participants and bias in research otherwise. However, despite these potential limitations, the SB tool had 16,000 users and 10,821 accounts created during the two-year intervention. Those numbers are to be placed in parallel with those from ORCHA (Organization for the Review of Care and Health Applications): more than 375,000 health applications were identified in 2020, 80% of them having less than 5000 downloads [105]. Both its uptake and the numerous publications in newspapers and magazines promoting it (over 30 articles) lead us to believe that SB reached an acceptable level of appropriateness and answered a real demand. Additional information on its relevance will be drawn from the full evaluation of its experimentation.

Such a tool is of interest only if users are aware of its existence, which means an adequate promotion strategy must be set up to reach the target population, regardless of its age, gender, and situation. On one hand, health promotion can be easily implemented at a national level by mobilizing available media sources, but this may be insufficient, as it risks being lost in the landscape of numerous health applications [30,106]. On the other hand, community-based health promotion (CBHP), and, in particular, city-level promotion [107], can provide an interesting framework to exploit in order to ensure the adoption of the tool on a smaller scale, adapted to the local population. First, it involves local actors in their own context, allowing them to use their own expertise and network. Moreover, by using local channels and collective experience, this approach can reach entire populations more efficiently [108]. Finally, it allows for aligning the promotion with individual characteristics, such as socioeconomic and environmental factors, key elements of mental health inequalities [108,109,110]. Nevertheless, reaching the entire target population remains difficult even with adapted tools, as local promotion, for example, will depend on local actors’ motivation and experience, and the allocated budget [108].

A city-level promotion was chosen for the promotion of SB, allowing the implementation of a two-year cluster-randomized controlled trial, which took place between April 2018 and March 2020, with a local promotion of the e-health tool by 42 local authorities initially involved in the project (ClinicalTrials.gov NCT03565562) [111]. GPs in particular were involved in the promotion through posters and flyers in their waiting rooms, and some seized the chance to address psychological distress and suicide with their patients using the tool (personal communication by Le Jeannic, 2022). This trial is now over and the evaluation, which is ongoing, will help us answer the question of the effectiveness of an e-health tool targeting the global population and its local promotion. 

## 5. Conclusions

Developing an e-health tool for suicide prevention offers many advantages when adapted to the need of the population. First, its accessibility allows different types of users to access the tool, especially hard-to-reach populations such as underage youth or the socially marginalized [60]. Second, its anonymity can reassure users, and the “online disinhibition effect" can reduce the shame related to the difficulty expressing emotions in front of others [112]. It is also a way to adapt health interventions to new cultural practices, as the Internet is now a widespread means to search for health information [113]. Finally, this technology allows many functions to be grouped in a single tool that can be used in the prevention of psychological distress without human interaction. But first and foremost, its functionalities can be an important part of users’ empowerment by helping them manage their illness on their own and gain autonomy, thereby improving their wellbeing [114,115,116].

Moreover, its promotion by the government itself during the pandemic shows that SB has its own place in the French landscape of e-health tools.

That being said, it is important to keep in mind that e-health tools should not be developed to make up for a potential lack in healthcare services but to be used as a reliable tool in the mental health care landscape, to help people evaluate themselves and be addressed to the healthcare system, if need be, at an early stage of their disease.

## Figures and Tables

**Table 1:** Selected mental health prevention programs.

Name	Country	Main Objective	Intervention
Optimizing Suicide Prevention Programs and their Implementation in Europe (OSPI Europe) [50,51]	Germany, Hungary, Ireland, and Portugal	To provide an evidence-based and efficient concept for suicide prevention along with the corresponding materials and instruments for the multifaceted intervention and guidelines for the implementation process in any European country.	The European Alliance Against Depression (EAAD) intervention consists of (1) training sessions and practice support for primary care physicians, (2) public relations activities and mass media campaigns, (3) training sessions for community facilitators who serve as gatekeepers for depressed and suicidal persons in the community and treatment, and (4) outreach and support for high risk and self-help groups (e.g., helplines). In OSPI, the EAAD model is enhanced by other evidence-based interventions and implemented simultaneously and in a standardized way in four regions.
European Regions Enforcing Actions against Suicide (Euregenas) www.euregenas.eu (accessed on 4 March 2023) [54,55]	Italy, Belgium, Sweden, Romania, Spain, Finland, Germany, Slovenia, United-Kingdom	To contribute to the prevention of suicidality (suicidal ideation, suicide attempts and suicide) in Europe through the development and implementation of technology-based strategies for suicide prevention at regional level. Those strategies can be of use to the European Community as examples of good practice.	WP 5: development of an e-conceptual model to provide all necessary information to be able to create an integrated support and intervention mainframe for e-mental health, directed at the prevention of suicide, which can be adapted to local needs in all European regions and regional health care organizations. WP 6: development of prevention guidelines and toolkits for suicide prevention strategies as well as specific prevention packages for the awareness raising on suicide prevention for the identified target groups.
Beyondblue [56,57]	Australia	To evaluate whether a campaign to increase public knowledge about depression (beyondblue: the national depression initiative) has influenced the Australian public’s ability to recognize depression and their beliefs about treatments.	A global campaign with a website: information depending on who you are; a chat; contact number or email; coping strategies for the user or a relative.
Preventing Depression and improving Awareness through Networking in the EU (PrediNu) [52,53]	Germany, Ireland, Belgium, Austria, Hungary, Bulgaria, Estonia, Portugal United-Kingdom, Spain and Luxembourg	To improve the care of depression and prevent suicidal behavior using information and communication technologies.	A multilingual information website about depression and suicidal behavior (www.ifightdepression.com) and a multilingual, internet-based self-management program for patients with minor, mild, and moderate forms of depression (the iFightDepression program) were developed. Based on the EAAD and OSPI projects.

**Table 2:** Description of characteristics of selected mobile health programs for suicide prevention.

Name	Country	Main Objective	Tool Structure	Outcomes
BackUp [60]	Belgium	To provide a free, easily accessible, independently usable application. It offers evidence-based tools to support a suicidal person coping with a crisis. Equally for people concerned about a suicidal person and want to help.	Tool to reach out; Hope box; Tool to identify coping strategies; Safety plan; Suicidal trigger; Coping strategies; Reach out to family, friends, and professionals; Create a safe environment.	Positive evaluation of the program. Self-help tools can have positive impact on suicidal ideation: (1) reach people not accessible by usual interventions, (2) contribute by being an addition to regular care.
MYPLAN [63,64,65]	Denmark	To propose an app-based safety plan to reduce suicidal ideation.	Safety plan; Suicidal trigger; Coping strategies; New strategies; Other strategies for inspiration; Distractions; Map with nearest ED; Prewritten messages to send; Direct phone links; Virtual hope box.	A mobile phone application could be useful for some populations, and the safety plan would help people interrupt an early suicidal process and have a feeling of empowerment.
ReliefLink [62,66]	United States	To create a reliable and user-friendly app designed to provide continuity and follow-up linkages for people at risk of committing suicide.	Mood tracking; Reminders for doctor’s appointments and medication; Safety plan (coping strategies); Location of the nearest hospitals and mental health treatment centers; Emergency button that can connect patients to helplines, providers, 911, and friends/family.	Won first prize (USD50,000) in the Suicide Prevention: Continuity of Care and Follow-up App Challenge sponsored by SAMHSA (Substance Abuse and Mental Health Services Administration).

**Table 3:** Questionnaires selected, for the user him/herself or to test a user’s relative.

Quiz Name	Questionnaire	Goal	Reason for Selection
To test yourself			
Psychological wellbeing Quiz	12-item General Health Questionnaire (GHQ-12) [79,80]	First screening on global mental health.	Chosen because of its few questions, and its stability and sensitivity to changes over a long period. Often used in general population survey.
Depression Quiz	Nine-item Patient Health Questionnaire (PHQ-9) [81,82,83]	To test the level of depression. To lighten the completion, it was only proposed to users with a GHQ-12 score > 2.	Chosen because of its few questions, and reliability and validity to test depression. It is also appreciated by users and does not have a saturation phenomenon.
Anxiety Quiz	Seven-item Generalized Anxiety Disorder (GAD-7) [84,85]	To test the level of anxiety. To lighten the completion, it was only proposed to users with a GHQ-12 score > 2.	Chosen because of its few questions, easiness, and reliability in testing anxiety.
Suicide risk evaluation	Mini International Neuropsychiatric Interview (MINI) [86,87]	Suicidality module for the level of suicidal risk. This questionnaire was proposed to users only from the moment: PHQ-9 score ≥ 10, or GAD-7 score > 7, or one score of the mood-tracking system was ≤40% or ≤50% with a 20% decrease in comparison to the previous score.	Chosen because of its few questions and reliability as a screening instrument more than to evaluate severity of symptoms.
To test a relative			
Depression Quiz	Montgomery and Asberg Depression Rating Scale (MADRS) [88]	To test relatives on depression and anxiety, it had to be hetero-administrated.	Chosen because it has only 10 questions and explores depression and anxiety with very good sensibility and sensitivity.

## Data Availability

The material generated and/or analyzed during the current study will be made available from the corresponding author (unique handler of the final dataset) on reasonable request within three years of the final collection of data.

## Abbreviations

CBHP	community-based health promotion
GP	general practitioner
INSERM	French National Institute of Health and Medical Research
SB	StopBlues
WHO	World Health Organization
GHQ-12	Twelve-item General Health Questionnaire
PHQ-9	Nine-item Patient Health Questionnaire
GAD-7	Seven-item Generalized Anxiety Disorder
SF-12	Twelve-item Short-Form Health Survey
MINI suicide	Mini International Neuropsychiatric Interview suicidality module

## Appendix A

**List A1.** Members of the PRINTEMPS Consortium.

The following are members of the PRINTEMPS Consortium: Corinne Alberti, Université de Paris, Unité UMR 1123 ECEVE, INSERM, Paris, France and Hôpital Robert Debré, CIC-EC, Unité INSERM CIC 1426, Assistance Publique-Hôpitaux de Paris, Paris, France; Karine Chevreul, Université de Paris, Unité UMR 1123 ECEVE, INSERM, Paris, France and Assistance Publique-Hôpitaux de Paris, Unité de Recherche Clinique en Économie de la Santé (URC Eco), 1 Place du Parvis Notre-Dame, 75004, Paris, France; Philippe Courtet, Department of Psychiatric Emergency and Acute Care, Lapeyronie Hospital, CHU Montpellier, Montpellier, France and Neuropsychiatry, Epidemiological and Clinical Research, INSERM, University of Montpellier, Montpellier, France; Coralie Gandré, Université de Paris, Unité UMR 1123 ECEVE, INSERM, Paris, France; Bruno Giraudeau, INSERM CIC 1415, CHRU de Tours, Tours, France and Tours University, Nantes University, INSERM SPHERE, U1246, Tours, France; Jean-Baptiste Hazo, Ministère des solidarités et de la santé, Drees, Paris, France; Anaïs Le Jeannic, Unité UMR 1123 ECEVE, INSERM, Paris, France and Assistance Publique-Hôpitaux de Paris, Unité de Recherche Clinique en Économie de la Santé (URC Eco), 1 Place du Parvis Notre-Dame, 75004, Paris, France; Jean-Luc Roelandt, World Health Organization Collaborating Centre for Research and Training in Mental Health, Établissement Public de Santé Mentale Lille Metropole, Lille, Hellemmes, France; Kathleen Turmaine, Université de Paris, Unité UMR 1123 ECEVE, INSERM, Paris, France; Guillaume Vaiva, Department of Adult Psychiatry, CHU Lille, Lille, France and Centre National de Ressources et Résilience pour le Psychotraumatisme (Cn2r Lille Paris), Lille, France; Marie-Amélie Vinet, Unité UMR 1123 ECEVE, INSERM, Paris, France and Assistance Publique-Hôpitaux de Paris, Unité de Recherche Clinique en Économie de la Santé (URC Eco), 1 Place du Parvis Notre-Dame, 75004, Paris, France.

**Table A1:** Search strategy in Embase.

#	Keywords	Number of Publications
1	‘suicide’/exp OR (‘suicide’/exp OR suicide:ab,ti AND (‘gesture’/exp OR gesture:ab,ti OR attempt:ab,ti OR ‘risk’/exp OR risk:ab,ti)) OR ‘suicidal ideation’/exp OR ‘suicidal ideation’:ab,ti OR (‘para suicidal’:ab,ti OR parasuicidal:ab,ti OR suicidal:ab,ti OR ‘suicide related’:ab,ti AND (‘behaviour’/exp OR behaviour:ab,ti OR ‘behavior’/exp OR behavior:ab,ti OR feelings:ab,ti OR thoughts:ab,ti)) OR ‘fatal self-harm’:ab,ti OR ‘fatal self harm’:ab,ti OR ‘fatal self injury’:ab,ti OR ‘fatal self-injury’:ab,ti OR ‘self-destructive:ab,ti and (behavior or behaviour):ab,ti’ OR ‘self destructive behaviour’:ab,ti OR ‘intentional life threatening behaviour’:ab,ti OR ‘intentional life threatening behavior’:ab,ti OR ‘self-cutting’:ab,ti OR ‘self cutting’:ab,ti OR ‘self inflicted wounds’:ab,ti OR ‘self-inflicted wounds’:ab,ti OR (deliberate:ab,ti AND (‘self cutting’:ab,ti OR ‘self poisoning’/exp OR ‘self poisoning’:ab,ti)) OR (‘self poisoning’/exp OR ‘self poisoning’:ab,ti AND (‘overdose’/exp OR overdose:ab,ti)) OR ‘drug overdose’/exp OR ‘drug overdose’:ab,ti OR ‘overdose’/exp OR overdose:ab,ti OR (‘non fatal’:ab,ti OR nonfatal:ab,ti OR fatal:ab,ti OR suicidal:ab,ti AND (‘self harm’/exp OR ‘self harm’:ab,ti OR ‘self injury’/exp OR ‘self injury’:ab,ti OR ‘self mutilation’/exp OR ‘self mutilation’:ab,ti OR ‘auto-mutilation’:ab,ti OR ‘automutilation’/exp OR automutilation:ab,ti)) OR ‘self injurious’:ab,ti OR ‘self-inflicted violence’:ab,ti OR ‘self intoxication’:ab,ti OR ‘self-directed violence’:ab,ti OR dsh:ab,ti OR sh:ab,ti OR si:ab,ti OR ‘sib’/exp OR sib:ab,ti OR ‘siv’/exp OR siv:ab,ti	528,631
2	‘web based’:ab,ti OR ‘internet based’:ab,ti OR ‘computer based’:ab,ti OR ‘cyber based’:ab,ti OR ‘internet mediated’:ab,ti OR ‘internet supported’:ab,ti OR ‘computer supported’:ab,ti OR ‘computer mediated’:ab,ti OR ‘web mediated’:ab,ti OR ‘web supported’:ab,ti OR ‘mobile health’:ab,ti AND (therapy:ab,ti OR intervention*:ab,ti OR prevention:ab,ti OR ‘prevention program’:ab,ti OR ‘prevention programme’:ab,ti OR ‘preventative tool’:ab,ti) OR ‘cyber intervention’:ab,ti OR ‘internet-supported therapeutic intervention’:ab,ti OR internet:ab,ti OR web:ab,ti OR technolog*:ab,ti OR online:ab,ti OR computer:ab,ti OR cyber*:ab,ti OR net:ab,ti OR virtual:ab,ti OR surf:ab,ti OR ‘electronic mail’:ab,ti OR email:ab,ti OR ‘e mail’:ab,ti OR forum:ab,ti OR ‘social media platform’:ab,ti OR site:ab,ti OR www:ab,ti OR eintervention:ab,ti OR ‘e intervention’:ab,ti OR ‘e therapy’:ab,ti OR etherapy:ab,ti OR ‘e health’:ab,ti OR ehealth:ab,ti OR mhealth:ab,ti OR (mobile:ab,ti AND health:ab,ti AND (app:ab,ti OR application:ab,ti))	1,613,911
3	#1 AND #2	20,700
4	Limit 3 to humans	10,392
5	#4 AND (‘camera’/de OR ‘computer’/de OR ‘computer system’/de OR ‘film’/de OR ‘mobile phone’/de OR ‘telephone’/de) AND (‘depression’/de OR ‘drug intoxication’/de OR ‘intoxication’/de OR ‘major depression’/de OR ‘mental disease’/de OR ‘schizophrenia’/de OR ‘suicidal behavior’/de OR ‘suicidal ideation’/de OR ‘suicide’/de OR ‘suicide attempt’/de) AND ‘human’/de	227
